# Development and External Validation of ^18^F-FDG PET-Based Radiomic Model for Predicting Pathologic Complete Response after Neoadjuvant Chemotherapy in Breast Cancer

**DOI:** 10.3390/cancers15153842

**Published:** 2023-07-28

**Authors:** Chae Hong Lim, Joon Young Choi, Joon Ho Choi, Jun-Hee Lee, Jihyoun Lee, Cheol Wan Lim, Zisun Kim, Sang-Keun Woo, Soo Bin Park, Jung Mi Park

**Affiliations:** 1 Department of Nuclear Medicine, Soonchunhyang University Seoul Hospital, Seoul 04401, Republic of Korea; 100819@schmc.ac.kr; 2 Department of Nuclear Medicine, Samsung Medical Center, Sungkyunkwan University School of Medicine, Seoul 06351, Republic of Korea; jynm.choi@samsung.com; 3 Department of Nuclear Medicine, Soonchunhyang University Bucheon Hospital, Bucheon 14584, Republic of Korea; 4 Department of Surgery, Soonchunhyang University Seoul Hospital, Seoul 04401, Republic of Korea; 5 Department of Surgery, Soonchunhyang University Bucheon Hospital, Bucheon 14584, Republic of Korea; 6 Division of Applied RI, Korea Institutes of Radiological and Medical Sciences, Seoul 01812, Republic of Korea

**Keywords:** breast cancer, ^18^F-fluorodeoxyglucose, PET/CT, pathologic complete response, radiomics

## Abstract

**Simple Summary:**

The pathologic complete response (pCR) after neoadjuvant chemotherapy (NAC) is a surrogate endpoint for predicting long-term clinical benefit in breast cancer. Recently, the use of radiomic features extracted from ^18^F-FDG PET/CT has emerged as a promising tool for predicting treatment outcomes in various cancers. We developed and externally validated a predictive model using ^18^F-FDG PET-based radiomics with the least absolute shrinkage and selection operator (LASSO) logistic method for pCR following NAC in breast cancer. Our radiomic-score model demonstrated satisfactory discriminative performances in training, internal validation, and external validation cohorts. Furthermore, the integrated radiomic model incorporating human epidermal growth factor receptor 2 (HER2) status showed improved performance compared to the radiomic-score model alone in all cohorts. The newly developed radiomic-score model might enable a more accurate and personalized assessment of the tumor response to neoadjuvant chemotherapy in breast cancer.

**Abstract:**

The aim of our retrospective study is to develop and externally validate an ^18^F-FDG PET-derived radiomics model for predicting pathologic complete response (pCR) after neoadjuvant chemotherapy (NAC) in breast cancer patients. A total of 87 breast cancer patients underwent curative surgery after NAC at Soonchunhyang University Seoul Hospital and were randomly assigned to a training cohort and an internal validation cohort. Radiomic features were extracted from pretreatment PET images. A radiomic-score model was generated using the LASSO method. A combination model incorporating significant clinical variables was constructed. These models were externally validated in a separate cohort of 28 patients from Soonchunhyang University Buscheon Hospital. The model performances were assessed using area under the receiver operating characteristic (AUC). Seven radiomic features were selected to calculate the radiomic-score. Among clinical variables, human epidermal growth factor receptor 2 status was an independent predictor of pCR. The radiomic-score model achieved good discriminability, with AUCs of 0.963, 0.731, and 0.729 for the training, internal validation, and external validation cohorts, respectively. The combination model showed improved predictive performance compared to the radiomic-score model alone, with AUCs of 0.993, 0.772, and 0.906 in three cohorts, respectively. The ^18^F-FDG PET-derived radiomic-based model is useful for predicting pCR after NAC in breast cancer.

## 1. Introduction

Neoadjuvant chemotherapy (NAC) is widely used to treat patients with locally advanced breast cancer (LABC) [1]. This approach can downstage breast cancer, increase rates of breast-conserving therapy (BCT), and minimize the need for aggressive nodal surgery [2]. Furthermore, some patients receiving NAC can achieve a pathologic complete response (pCR), meaning that all tumor cells have been eradicated. In the last decade, studies have proposed pCR after NAC as a surrogate endpoint for predicting long-term clinical benefit, such as disease-free survival and overall survival [3,4]. In the future, they might also have the chance of omitting surgery [5]. Therefore, there is an increasing demand to develop a more reliable diagnostic method to accurately predict pCR after NAC.

Anatomical imaging modalities, such as magnetic resonance imaging (MRI), have traditionally played a crucial role in breast cancer staging and assessment. However, they primarily rely on visualizing changes in tumor size and morphology, which may not fully capture the complex alterations in tumor biology. The lack of comprehensive metabolic and functional data from anatomical imaging can lead to suboptimal prediction of pCR and potentially limit the accuracy of the treatment response assessment [6,7].

^18^F-fluorodeoxyglucose positron emission tomography/computed tomography (^18^F FDG PET/CT) is a useful imaging modality for staging and restaging in breast cancer [8]. ^18^F-FDG PET/CT can also provide more information on tumor biology in evaluating breast cancer than conventional anatomic imaging modalities [9,10,11]. Many studies have reported the clinical utility of metabolic parameters measured on ^18^F-FDG PET/CT for predicting therapeutic response and survival outcomes [12,13]. Furthermore, the use of texture analysis, currently known as “radiomics”, to derive a large amount of quantitative parameters has improved the predictive power in the field of oncology [14].

Recent studies have investigated the potential of a radiomics model using texture parameters extracted from ^18^F-FDG PET/CT in predicting pCR after NAC in patients with breast cancer [15,16,17]. However, it is still challenging to achieve optimal performance and generalizability of the model due to investigators’ failure to use classification methods that are appropriate for high-dimensional data or perform an external validation test [18]. The least absolute shrinkage and selection operator (LASSO) method has been widely used for analyzing high-dimensional data [19]. Thus, the aim of this study was to develop an ^18^F-FDG PET-derived radiomics model using the LASSO method for predicting pCR after NAC in breast cancer and externally validate it.

## 2. Patients and Methods

### 2.1. Study Population

**Figure 1:**
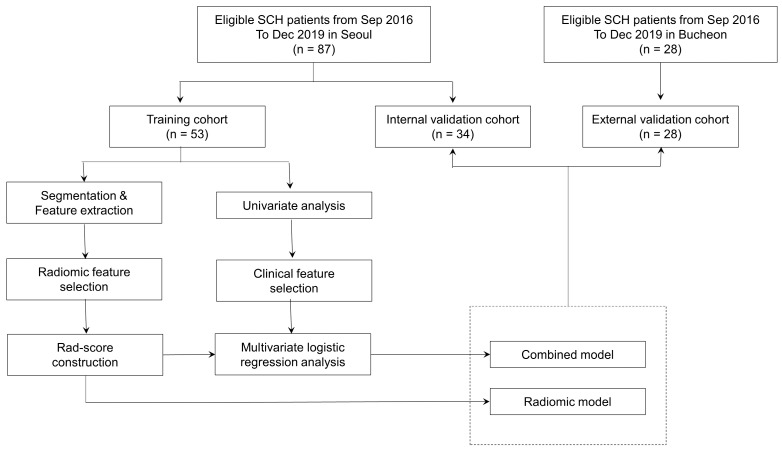
Study workflow. SCH, Soonchunhyang University Hospital.

This study was approved by our Institutional Review Board (IRB). The requirement for informed consent was waived by the IRB due to its retrospective nature. We conducted a retrospective review of medical records of consecutive breast cancer patients who underwent pretreatment with ^18^F-FDG PET/CT for initial staging at Soonchunhyang University Seoul and Bucheon Hospitals between September 2016 and December 2019. Inclusion criteria were: (1) female sex, (2) pathologically-proven invasive ductal carcinoma, (3) clinical stage II-III, and (4) receiving curative surgery after completing NAC. The NAC regimens consisted of four cycles of Adriamycin and Cyclophosphamide (AC), followed by four cycles of Taxotere (T). Additionally, some patients received four cycles of weekly Paclitaxel and Carboplatin, followed by four cycles of AC. Patients with human epidermal growth factor receptor 2 (HER2) amplification received six cycles of Taxotere, Carboplatin, Herceptin, and Perjeta Exclusion criteria were: (1) a tumor with inadequate metabolic activity that could not be delineated with an SUV cut-off of 2.5, and (2) multifocal or multicentric breast cancer. Eligible patients from Soonchunhyang University Seoul Hospital (SCH, Seoul, Republic of Korea) were included in either a training cohort or an internal validation cohort. Those from Soonchunhyang University Bucheon Hospital (SCH, Bucheon, Republic of Korea) were enrolled in an external validation cohort. The study workflow is presented in Figure 1.

### 2.2. Data Collection

All clinicopathologic data were collected from electronic medical records. Clinical data included age at initial diagnosis, the American Joint Committee on Cancer (AJCC) TNM stage, tumor location, surgery method, and tumor marker (cancer antigen 15-3). Pathologic data included estrogen receptor (ER), progesterone receptor (PR), HER2, and pCR. ER, PR, and HER2 expression data were collected from reports of biopsies performed before initiation of NAC. ER and PR positivity were defined as at least 1% of nuclear staining in tumor cells. HER2 IHC was scored as positive (3+), equivocal (2+), or negative (1+/0). HER2 status was considered positive if an immunohistochemical (IHC) test score of 3+ was recorded or if there was positive gene amplification using in situ hybridization testing. Patients with an IHC score of 2+ were tested for HER2 amplification by FISH. Pathologic CR was evaluated using a surgical specimen following completion of NAC. It was defined as the absence of any remaining invasive disease or the presence of residual ductal carcinoma in situ without any remaining lymph node metastasis [4].

### 2.3. ^18^F-FDG PET/CT Image Acquisition and Analysis

All patients were instructed to fast for at least 6 h before undergoing PET/CT scans to maintain their blood glucose level below 200 mg/dL. PET/CT images at SCH, Seoul were acquired using a PET/CT scanner (Biography 128 mCT, Siemens Healthcare, Erlangen, Germany). A non-enhanced CT scan was conducted 60 min after administering 4.44 MBq/kg of ^18^F-FDG using a 128-slice spiral CT scanner (100 keV; 65 mAs with Auto Care Dose; section width 3.0 mm). Three-dimensional emission PET data were acquired from the thigh to the head for each frame, with a duration of 2.5 min. The PET images underwent reconstruction using CT for attenuation correction, utilizing the TrueX + TOF method provided by the manufacturer (21 subsets, 2 iterations). The image matrix size was 400 × 400 with a voxel size of 2.03 × 2.03 × 3.0 mm^3^. At the SCH in Bucheon, PET/CT scans were performed using a PET/CT scanner (Biograph 128 mCT, Siemens Medical Solutions, Knoxville, TN, USA). Similar to the first site, an unenhanced CT scan was performed 60 min after injecting 4.07 MBq/kg of 18F-FDG with a 128-slice spiral CT scanner (100 keV; 65 mAs with Auto Care Dose; section width 3.0 mm). Three-dimensional emission PET data were acquired from the thigh to the head for each frame, with a duration of 2.5 min. The PET images were reconstructed using CT for attenuation correction, employing the TrueX + TOF method offered by the manufacturer (21 subsets, 2 iterations). The image matrix was 200 × 200 with a voxel size of 4.07 × 4.07 × 3.0 mm^3^. The volume of interest (VOI) for the breast lesion was delineated on PET images using a threshold of 2.5 of the maximum standardized uptake value (SUVmax) in MIM version 6.4 (MIM Software Inc., Cleveland, OH, USA).

### 2.4. ^18^F-FDG Radiomic Feature Extraction

^18^F-FDG PET/CT radiomic features were extracted from segmented tumors on PET images using the Chang-Gung Image Texture Analysis “CGITA” software package (http://code.google.com/archive/p/cgita (accessed on 12 May 2021)) [20]. It is a freeware and open-source software developed in Matlab for quantifying tumor heterogeneity with molecular images. A total of 72 radiomic features were calculated and grouped into several categories (Supplemental Table S1). These categories included co-occurrence (6 features), voxel alignment (11 features), Neighborhood Intensity Difference (NID) (5 features), Intensity Size-Zone (ISZ) (11 features), Normalized Co-occurrence (7 features), voxel statics (13 features), texture spectrum (2 features Texture Feature Coding (TFC) (4 features), Texture Feature Coding Co-occurrence (TFCC) (8 features), and Neighborhood Gray-Level Dependence (NGLD) (5 features). To mitigate the influence of PET image acquisition and reconstruction factors, specifically scanner effects, on imaging parameters, we implemented a modified version of the ComBat harmonization method known as M-ComBat [21,22]. This approach enabled us to align the radiomic feature distributions of the external validation data with the mean and variance of the training data, serving as the reference center.

### 2.5. ^18^F-FDG Radiomic Feature Selection and Model Construction

The primary cohort (SCH, Seoul, Republic of Korea) was randomly divided into a training set and an internal validation set. The training set was used to construct a predictive model for pCR after completion of NAC. To build the final model, we used the LASSO algorithm to select an optimized subset of features through regularization. Prior to feature selection and model building, no data transformation or standardization was conducted. In LASSO regression, the tuning parameter lambda (λ) controls the amount of regularization applied to the model. When lambda is large, coefficients for variables with smaller absolute values are compressed to zero. We applied a 10-fold cross-validation method to identify the optimal value of λ, which minimized the mean cross-validation error. The variables with non-zero coefficients at the optimal λ were considered the most predictive radiomic features. The radiomic score was calculated as the sum of the selected radiomic features multiplied by their corresponding non-zero coefficients identified by the optimal λ [23,24]. This was referred to as the radiomic-score model. Subsequently, we performed univariate and multivariate logistic regression analyses to identify the most useful clinical variables for prediction. We then built a multivariate logistic prediction model by combining the radiomic score and selected clinical variables. This was referred to as the combination model.

The performance of each model was evaluated using area under the receiver operating characteristic (ROC) curve (AUC) with 95% confidence intervals (CIs). Differences in AUC values among models were assessed using the DeLong test. Calibration curves were constructed to assess the agreement between predicted probabilities and observed outcomes. The Hosmer–Lemeshow test was used to determine the goodness of fit. A *p*-value of greater than 0.05 indicated good calibration.

### 2.6. Statistical Analysis

All statistical analyses were performed using open source software R version 3.6.1 (The R Foundation for Statistical Computing, Vienna, Austria) and MedCalc 15.5 (MedCalc, Mariakerke, Belgium). The primary cohort in SCH, Seoul was randomly split using a conservative method with the “caret” package, and LASSO logistic regression was performed using the glmnet package. M-ComBat correction was applied using the “SVA” package. The “ResourceSelection” package was used for calibration curve analysis. Categorical variables were compared using Chi-square or Fisher exact tests for categorical variables. Continuous variables were compared with the Mann–Whitney U-test or Kruskal–Wallis test. All tests were two-sided, and statistical significance was set at *p* < 0.05.

## 3. Results

### 3.1. Baseline Characteristics of Patients

A total of 82 female breast cancer patients who received neoadjuvant chemotherapy were included in training and internal validation cohorts, and 28 patients were enrolled in the external validation cohort. These patients ranged in age from 27 to 70 years old. The clinical characteristics of patients in training and validation cohorts are summarized in Table 1. Of patients included in this study, 10 (18.9%, 10/53), five (14.7%, 5/34), and four (14.3%, 4/28) cases achieved pCR after completing neoadjuvant chemotherapy in the training cohort, internal validation cohort, and external validation cohort, respectively. However, the difference was not statistically significant (*p* = 0.821). Baseline levels of CA15-3 were significantly higher in the external validation cohort than in the training cohort and internal validation cohort (*p* = 0.001). Otherwise, there were no significant differences in other clinical variables between cohorts.

### 3.2. Comparison of Clinical Variables and Conventional PET Parameters According to pCR

We compared clinical variables and conventional PET parameters between the groups with pCR and non-pCR in the training cohort (Table 2). The occurrence of pCR was found to be significantly higher in HER2-positive tumors (*p* < 0.001). However, no other clinical variables showed significant differences between the two groups. Regarding the conventional PET parameters, the SUVmax was higher in the group with pCR, while the MTV and TLG were higher in the non-pCR group. However, none of these differences reached statistical significance.

### 3.3. ^18^F-FDG Radiomic Feature Selection and Model Construction

**Figure 2:**
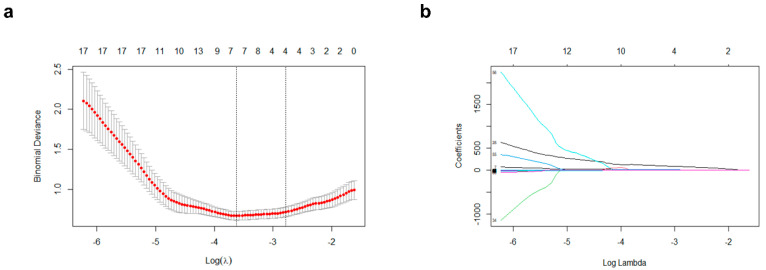
Radiomic feature selection using the least absolute shrinkage and selection operator (LASSO) logistic regression model. (**a**) Identification of the optimal penalization coefficient lambda (λ) in the LASSO model used 10-fold cross-validation and the minimum criterion. As a result, a λ value of 0.026817 was selected. (**b**) LASSO coefficient profiles of 7 features selected among 72 features.

Using the LASSO logistic regression method with a ten-fold cross-validation, seven radiomics features were selected from a total of 72 features to calculate the radiomics score for each patient (Figure 2). The radiomic score was calculated using a simple linear combination of seven selected indicators multiplied by their respective non-zero coefficients [23,24], as follows:

Radiomic score = (121.0130 ∗ Low-intensity zone emphasis) + (13.5401 ∗ TFCC_Inverse difference moment) + (10.9128 ∗ Short-run emphasis) + (3.4582 ∗ Max spectrum) + (−0.4898 ∗ TFCC_Code Entropy) + (0.0884 ∗ Strength) + (−0.0324 ∗ TFCC_Entropy)

Both univariate and multivariate logistic regression analyses were conducted to evaluate associations among the radiomic score, clinical variables, and pCR in the training cohort (Table 3). Results of univariate analysis demonstrated that the radiomic score (*p* < 0.001) and HER2 status (*p* = 0.003) were significantly associated with pCR (Table 2). In the multivariate analysis, both the radiomic score (*p* = 0.022) and HER2 status (*p* = 0.049) were identified as independent predictors of pCR. The logistic regression model including these two variables yielded predicted probabilities for achieving pCR after NAC.

### 3.4. Model Performance and Validation

**Figure 3:**
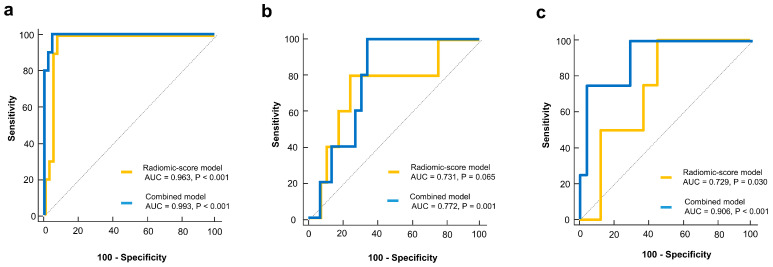
ROC curves of radiomic and combined models. (**a**) Training cohort, (**b**) Internal validation cohort, and (**c**) External validation cohort.

**Figure 4:**
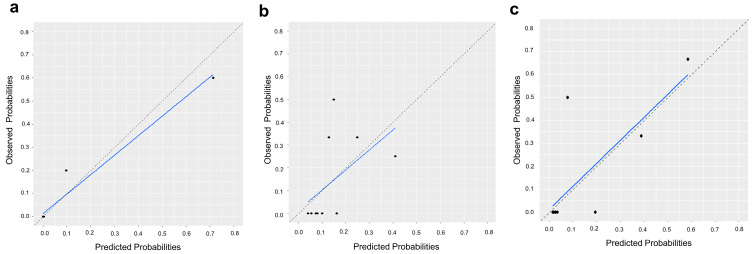
Calibration curves of combined model. (**a**) Training cohort, (**b**) Internal validation cohort, and (**c**) External validation cohort. The blue line indicates the actual incidence of pCR. The dashed line indicates the predicted incidence of pCR.

The radiomic-score model demonstrated excellent discriminative performance for predicting pCR in the training cohort (AUC: 0.963, 95% CI: 0.871 to 0.996). The radiomics model achieved satisfactory discrimination in internal and external validation cohorts, with AUCs of 0.731 (95% CI: 0.552 to 0.868) and 0.729 (95% CI: 0.529 to 0.878), respectively (Figure 3). The combination model showed improved predictive performance compared to the radiomic-score model alone, with AUCs of 0.993 (95% CI: 0.920 to 1.000), 0.772 (95% CI: 0.597 to 0.898), and 0.906 (95% CI: 0.735 to 0.983) in the training, internal validation, and external validation cohorts, respectively (Figure 3). The calibration curve of the combination model also revealed good agreement between the observed outcome and prediction in all three cohorts (Figure 4). Additionally, the Hosmer–Lemeshow test yielded a non-significant statistic in all three cohorts (*p* = 0.998, *p* = 0.501, and *p* = 0.618, respectively), indicating that the model fit well.

## 4. Discussion

Achievement of pCR after NAC has been proposed as a surrogate endpoint for predicting long-term clinical benefit in breast cancer patients [3,4]. In this regard, studies have tried to develop biomarkers for predicting pCR using various medical data. Recently, the use of radiomic features extracted from ^18^F-FDG PET/CT has emerged as a promising tool for predicting treatment outcomes in various cancers [25,26]. Therefore, we developed and validated a predictive model using ^18^F-FDG PET-based radiomics with the LASSO method for pCR following NAC in breast cancer. Our radiomic-score model demonstrated satisfactory discriminative performances in the training, internal validation, and external validation cohorts. Furthermore, the integrated radiomic model incorporating the HER2 status showed improved performance compared to the radiomic-score model alone in all cohorts. 

Some studies have explored the predictive model using ^18^F-FDG-based radiomics for pCR after NAC in breast cancer patients. Lee et al. constructed a clinical model using ^18^F-FDG radiomic features [17]. However, this study demonstrated a suboptimal performance for predicting pCR (AUC = 0.623 in the training split set and AUC = 0.640 in the independent validation set). Li et al. developed a model using baseline ^18^F-FDG PET/CT derived radiomic features and revealed a good performance to predict pCR prior to NAC (AUC = 0.844 in the training split set and AUC = 0.722 in the independent validation set) [15]. However, the lack of model calibration and external validation may limit the generality and robustness of their findings [27]. Our model exhibited excellent performance for predicting pCR in the training cohort (AUC = 0.963). It also showed satisfactory performances in the two validation cohorts (AUC = 0.731 in the internal validation set and AUC = 0.729 in the external validation set). Additionally, our model was well-calibrated in calibration analysis.

Conventional ^18^F-FDG parameters such as maximum SUV, MTV, and TLG reflect the metabolic activity and burden within tumor cells. Many studies have reported the usefulness of these parameters as predictive biomarkers for treatment outcome and survival prognosis in various cancers [28,29,30]. Some studies have reported that a high SUVmax or TLG may help predict pCR after completion of NAC in breast cancer patients [31,32]. However, in our study, these parameters were not included in significant features for predicting pCR. Unlike previous research, which mainly focused on hormone-positive breast cancer, our study included a broader range of breast cancer subtypes, such as HER2, TNBC, and hormone-positive tumors. This difference in population contributed to the contrasting results. Furthermore, our findings suggest that conventional parameters may have limitations in assessing intra-tumor heterogeneity, a crucial factor in predicting therapeutic resistance in breast cancer [33].

Intra-tumor heterogeneity of ^18^F-FDG uptake can potentially be quantified with textural features extracted from obtained PET images through complex mathematical models of the relationship between multiple image voxels [34]. Such texture parameters are classified into first-order features and higher-order features. First-order features describe the overall distribution of voxel intensities in the image, while higher-order features describe spatial relationships between the voxels’ intensities. In the present study, seven higher-order features including low-intensity zone emphasis, short-run emphasis, max spectrum, strength, and three TFCC features were selected as significant relevant features with pCR following NAC in breast cancer. Some studies have also reported that higher-order ^18^F-FDG texture features have closer associations with achievement of pCR following NAC in breast cancer [15,16,17]. These findings may suggest that achieving pCR of breast cancer following NAC is linked to spatial heterogeneity of tumor cell metabolism [35,36]. However, further research is needed to directly investigate the association between these features and tumor biology to gain a better understanding of this relationship and its potential clinical implications.

The use of texture analysis in PET/CT images can yield a very large number of parameters that can be theoretically calculated [37]. In high-dimensional data, selecting appropriate features plays an essential role in improving the discriminative power of predictive models. Previous studies have employed logistic regression analysis to select significant ^18^F-FDG texture features to predict pCR following NAC in breast cancer [16,17]. However, this method may cause multiple testing issues, increasing the risk of false-positive findings when testing many hypotheses [38]. Recently, LASSO regression has been used extensively in radiomics studies to reduce data dimensions and multicollinearity among features [19]. Therefore, we constructed the predictive model using seven textural features selected by LASSO regression in the training cohort. Furthermore, this method contributes to improving the predictive performance in the validation cohort by minimizing overfitting in the training cohort. However, despite using the LASSO method, our predictive model showed some validation loss, which might be attributed to the small sample size of the validation set. Our results should be validated in future studies with larger populations.

Although there is a wide range of tumor delineation methods available, the optimal segmentation method for PET radiomics research remains a topic of debate. In our study, we utilized a threshold-based segmentation method, specifically using SUV 2.5, to delineate breast lesions on PET images. The use of a cutoff value of SUV 2.5 is a commonly employed fixed thresholding approach for malignant tumor delineation [39,40,41]. Additionally, the fixed threshold method using SUV 2.5 demonstrates superior inter-observer agreement compared to other threshold methodologies [42]. This method allows for easy reproducibility, as the same threshold value can be consistently applied for clinical utility and the development of the predictive model. However, it is important to acknowledge that different tumor delineation methods can impact radiomic feature values [43]. In future studies, it is essential to investigate the influence of segmentation methods on our results to gain a comprehensive understanding of their potential implications.

External validation is crucial in prediction model research to evaluate the reliability and generalizability of the developed model. However, there is a lack of studies that have externally validated predictive models using ^18^F-FDG-derived radiomics for pCR in breast cancer. Therefore, we conducted external validation of our predictive model using independent data from a different center. Radiomic features are sensitive to differences between centers caused by scanner models, acquisition protocols, and reconstruction settings, known as the “center effect” [44]. To address this, we used a modified version of the ComBat harmonization method called M-ComBat [21]. M-ComBat allows for flexible and robust adjustment of data to a specific reference center, overcoming the limitations of traditional ComBat. Recent research supports the effectiveness of M-ComBat in harmonizing data from different centers [22,45]. By employing M-ComBat, we addressed the potential confounding effects arising from scanner variations and ensured a consistent and standardized comparison across datasets. Our predictive model maintained high performance even when applied to this independent dataset, further validating its reliability.

Our study also examined clinical variables associated with pCR following NAC in patients with breast cancer. Consistent with previous studies [34], we found that HER2-positive status was strongly associated with achieving pCR. Additionally, integrating HER2 status into a radiomic model significantly improved the model’s predictive performance for pCR after NAC. However, hormone receptor status, TNBC, CA15-3 level, tumor stage, and age did not demonstrate a significant association with achieving pCR following NAC. Although some previous studies have investigated Ki67 expression as a potential predictor of pCR after NAC [35], it was not included in our analysis. At our institution, Ki67 was not routinely assessed in the pretherapeutic stage due to its limited value for treatment decision-making and questionable analytical validity [36]. Consequently, our finding suggests that HER2 status is an important clinical variable in predicting pCR after NAC, and radiomic models incorporating HER2 status might be useful in improving predictive performance.

The limitations of this study include inherent biases due to the retrospective design with the limited and unbalanced sample size. In addition, this study has some limitations in terms of methodology. First, CGITA used for the feature extraction does not comply completely with all the recommendations of the Imaging Biomarker Standardization Initiative (IBSI). This represents a clear limitation from the perspective of standardization in radiomics research. However, the clinical utility of the extracted PET radiomic features from CGITA has been validated in numerous studies, including breast cancer research [41,46,47,48]. Future research should include comparisons with features presented by IBSI. Additionally, some radiomic features may be influenced by variations in tumor volume. Partial-volume effects, particularly in smaller lesions, can increase heterogeneity due to the limited spatial resolution of PET scanners [49,50]. A previous study included only lesions with a volume greater than 1.5 cm^3^ to mitigate the impact of partial-volume effects considering the spatial resolution of the scanner [51]. Another study on ^18^F-FDG radiomics strictly included subjects based on a minimum volume criterion of 4.2 cm^3^ [52]. In our study, all tumors had a volume larger than 1.5 cm^3^, with the majority (90%) exceeding 4.2 cm^3^. However, further investigations considering changes in tumor volume are necessary to gain deeper insights into this issue.

## 5. Conclusions

An ^18^F-PET-based radiomic model using the LASSO algorithm exhibited good performance in predicting pCR following NAC in breast cancer. Furthermore, the combined model incorporating HER2 status showed improved performance compared to the radiomic model alone. It might enable a more accurate and personalized assessment of the tumor response to neoadjuvant chemotherapy in breast cancer. Further prospective validation studies are needed to confirm the practical applicability of this potential imaging biomarker.

## Figures and Tables

**Table 1:** Clinical characteristics of the training cohort and the validation cohorts.

Variable	Training (*n* = 53)	Internal Validation (*n* = 34)	External Validation (*n* = 28)	*p* Value
Age	49.5 ± 10.8	49.7 ± 9.6	48.7 ± 9.8	0.929
Clinical tumor stage				0.190
II	23 (43.4)	9 (26.5)	13 (46.4)	
III	30 (56.6)	25 (73.5)	15 (53.6)	
Receptor status in histology				0.812
ER-positive	33 (62.3)	19 (55.9)	15 (53.6)	
PR-positive	29 (54.7)	18 (52.9)	10 (35.7)	
HER2-postive	16 (30.2)	7 (20.6)	7 (25.0)	
TNBC	11 (20.8)	9 (26.5)	9 (32.1)	
Surgery				0.239
Breast conserving surgery	12 (22.6)	13 (38.2)	10 (35.7)	
Mastectomy	41 (77.4)	21 (61.8)	18 (64.3)	
Baseline CA15-3	9.6 ± 4.5	9.7 ± 4.5	18.8 ± 11.7	0.001
Tumor location				0.155
Right	28 (52.8)	11 (32.4)	14 (50.0)	
Left	25 (47.2)	23 (67.6)	14 (50.0)	
Response to NAC				0.821
pCR	10 (18.9)	5 (14.7)	4 (14.3)	
Non-pCR	43 (81.1)	29 (85.3)	24 (85.7)	

Data are presented as numbers (%) or mean ± standard deviation. ER, estrogen receptor; PR, progesterone receptor; HER2, human epidermal growth factor receptor 2; TNBC, triple-negative breast cancer; CA, cancer antigen; NAC, neoadjuvant chemotherapy; pCR, pathologic complete response.

**Table 2:** Comparison of clinical variables and conventional PET parameters between groups with pCR and non-pCR in the training cohort.

Variable	pCR (*n* = 10)	Non-pCR (*n* = 43)	*p* Value
Age	52.8 ± 10.4	48.7 ± 10.8	0.223
Clinical tumor stage			0.484
II	3 (30.0)	20 (46.5)	
III	7 (70.0)	23 (53.5)	
Receptor status in histology			
ER-positive	5 (50.0)	28 (65.1)	0.475
PR-positive	3 (30.0)	26 (60.5)	0.156
HER2-postive	8 (80.0)	8 (18.6)	<0.001
TNBC	1 (10.0)	10 (23.3)	0.667
Surgery			0.207
Breast conserving surgery	4 (40.0)	8 (18.6)	
Mastectomy	6 (60.0)	35 (81.4)	
Baseline CA15-3	8.1 ± 3.5	9.9 ± 4.6	0.285
Tumor location			0.999
Right	5 (50.0)	23 (53.5)	
Left	5 (50.0)	20 (46.5)	
Conventional PET parameter			
SUVmax	12.2 (6.4–19.6)	8.3 (5.5–15.1)	0.328
MTV	8.2 (4.2–11.4)	12.3 (6.0–22.7)	0.112
TLG	25.1 (11.7–40.5)	38.3 (18.2–112.9)	0.228

Data are presented as numbers (%), mean ± standard deviation, or median (interquartile range). pCR, pathologic complete response; ER, estrogen receptor; PR, progesterone receptor; HER2, human epidermal growth factor receptor 2; TNBC, triple-negative breast cancer; CA, cancer antigen; PET, positron emission tomograpy; SUVmax, maximum standardized uptake value; MTV, metabolic tumor volum; TLG, total lesion glycolysis.

**Table 3:** Univariate and multivariate logistic regression analyses for clinical characteristics and radiomics score in the training cohort.

Variables	Univariate Logistic Analysis		Multivariate Logistic Analysis	
	OR (95% CI)	*p* Value	OR (95% CI)	*p* Value
Age	1.04 (0.97–1.11)	0.273	-	
CA15-3	0.90 (0.74–1.08)	0.799	-	
Stage	0.49 (0.11–2.16)	0.335	-	
ER positive	1.80 (0.45–7.23)	0.576	-	
PR positive	3.43 (0.78–15.17)	0.193	-	
HER2 positive	8.82 (1.89–41.09)	0.003	433.82 (1.03–182,988.53)	0.049
TNBC	0.37 (0.04–3.26)	0.320	-	
Radiomics score	9.71 (2.01–46.91)	<0.001	38.33 (1.70–866.00)	0.022

OR, odds ratio; CI, confidence interval; CA, cancer antigen; ER, estrogen receptor; PR, progesterone receptor; HER2, human epidermal growth factor receptor 2; TNBC, triple-negative breast cancer.

## Data Availability

The datasets generated during and/or analyzed during the current study are available from the corresponding author on reasonable request.

## Supplementary Materials

The following supporting information can be downloaded at: https://www.mdpi.com/article/10.3390/cancers15153842/s1, Table S1: List of 72 Quantitative PET-Based Radiomic Features.
