# Supplementary material for: Development and External Validation of 18F-FDG PET-Based Radiomic Model for Predicting Pathologic Complete Response after Neoadjuvant Chemotherapy in Breast Cancer
Source: Cancers (Basel). 2023 Jul 28;15(15):3842. doi: 10.3390/cancers15153842 (PMC10417050; doi:10.3390/cancers15153842)
Supplement: Supplementary file 1 [file cancers-15-03842-s001.zip › cancers-2506491-supplementary.pdf]

Table S1. List of 72 Quantitative PET-Based Radiomic Features.

Parent matrix	Radiomic features
Co-occurrence	Second angular moment, contrast, entropy, homogeneity, dissimilarity, inverse difference moment
Voxel-alignment	Short-run emphasis, long-run emphasis, intensity variability, run-length variability, run percentage, low-intensity run emphasis, high-intensity run emphasis, low-intensity short-run emphasis, high-intensity short-run emphasis, low-intensity long-run emphasis, high-intensity long-run emphasis
Neighborhood intensity difference	Coarseness, contrast, busyness, complexity, strength
Intensity size-zone	Short-zone emphasis, large-zone emphasis, intensity variability, size-zone variability, zone percentage, low-intensity zone emphasis, high-intensity zone emphasis, low-intensity short-zone emphasis, high-intensity short-zone emphasis, low-intensity large-zone emphasis, high-intensity large-zone emphasis
Normalized co-occurrence	Second angular moment, contrast, entropy, homogeneity, inverse difference moment, dissimilarity, cooccurrence correlation
Voxel statics	Minimum SUV, maximum SUV, mean SUV, SUV variance, SUV SD, SUV skewness, SUV kurtosis, SUV skewness (bias corrected), SUV kurtosis (bias corrected), TLG, tumor volume, entropy, SULpeak
Texture spectrum	Max spectrum, Black-white symmetry
Texture feature coding	Coarseness, homogeneity, mean convergence, variance
Texture feature coding cooccurrence matrix	Second angular moment, contrast, entropy, homogeneity, intensity, inverse difference moment, code entropy, code similarity
Neighborhood gray-level dependence	Small-number emphasis, large-number emphasis, number nonuniformity, second moment, entropy

SUV, standardized uptake value; SD, standard deviation; TLG, total lesion glycolysis.
